# Sex-Related Effects of Adrenergic Drugs on Conditioned Pain Modulation: A Randomized Controlled Cross-Over Double-Blind Trial

**DOI:** 10.1155/2022/2757101

**Published:** 2022-10-26

**Authors:** Marie-Philippe Harvey, Marie-Chantal Dubois, Philippe Chalaye, Yanick Sansoucy, Serge Marchand

**Affiliations:** ^1^Research Center on Aging, CIUSSS de l'Estrie-CHUS, Sherbrooke, Québec, Canada; ^2^Faculty of Medicine and Health Sciences, Sherbrooke University, Sherbrooke, Québec, Canada; ^3^Department of Anesthesiology, Faculty of Medicine and Health Sciences, Sherbrooke University, Sherbrooke, Québec, Canada; ^4^Department of Surgery, Faculty of Medicine and Health Sciences, Sherbrooke University, Sherbrooke, Québec, Canada; ^5^CSO–Lucine, Nouvelle-Aquitaine, Bordeaux, France

## Abstract

**Objective:**

Endogenous pain inhibition can be investigated using conditioned pain modulation (CPM). CPM efficacy has been reported to be influenced by various factors, such as gender and cardiovascular (autonomic) activity. The aim of this study is to describe the effect of pharmacological manipulations of autonomic activity on CPM efficacy.

**Methods:**

Thirty healthy participants were enrolled to assess CPM efficacy in 4 experimental sessions. The first session consisted of the determination of baseline CPM effectiveness. The three following sessions were performed in a randomized order and consisted of the injection of (1) esmolol, (2) ephedrine, or (3) placebo, before the conditioning stimulus. Pain intensity induced by using a contact heat stimulation thermode was compared before and after a cold-pressure conditioning stimulus to evaluate CPM effectiveness.

**Results:**

Our results show that inhibiting sympathetic nervous activity with esmolol did not have a significant effect on CPM. Conversely, enhancing sympathetic nervous activity with ephedrine increased CPM effectiveness in healthy women but decreased it in men.

**Conclusions:**

Increasing sympathetic activity with adrenergic agonists, such as ephedrine, could improve CPM effectiveness in women. It will be interesting to verify if the same results are present in patients suffering from chronic pain and if adrenergic agonists could have better therapeutic effects in women showing reduced CPM effectiveness.

## 1. Introduction

Pain is influenced by endogenous modulatory mechanisms [1, 2]. Descending pain inhibitory mechanisms, such as diffuse noxious inhibitory control, have been widely investigated in animals and humans [3, 4]. More precisely, diffuse noxious inhibitory control involves descending inhibitory pathways from the brainstem that diffusely reduce nociceptive activity [5, 6].

Diffuse noxious inhibitory control mechanisms have been associated with the “pain inhibits pain” or counter-irritation phenomenon. In clinical settings, the effectiveness of descending endogenous pain inhibitory mechanisms is commonly evaluated with the conditioned pain modulation (CPM) paradigm [7]. A strong noxious stimulus, referred to as the conditioning stimulus (CS), is used to activate descending pain inhibition [8]. One of the most commonly used CS is the cold pressor test (CPT), which consists of the immersion of the arm in cold water. The pain intensity induced by a painful test stimulus (TS) is compared before and after (or simultaneous) administration of the CS to determine the effectiveness of CPM. Several factors (age, sex, and pain intensity caused by the CS) have been associated with CPM effectiveness [9, 10].

A dysfunction in CPM activation has been reported in many patients suffering from chronic pain conditions such as fibromyalgia [11], irritable bowel syndrome [12], and temporomandibular disorder [13, 14]. A higher prevalence of chronic pain conditions is generally observed in women compared to men [15]. Interestingly, many investigators observed gender differences in CPM effects, with women showing less effective CPM compared to men [9, 16–20]. However, some studies reported no important difference in CPM effectiveness between sex [21, 22].

The effectiveness of CPM has also been associated with cardiovascular responses to pain [11, 12]. More specifically, greater sympathetic activity seems to be related to more important pain inhibition. Furthermore, different brainstem regions (reticular formation and periaqueductal gray) involved in descending inhibition are also known to regulate autonomic cardiovascular activity [23, 24]. Nociceptive input could trigger CPM and the cardiovascular responses induced by pain by activating the same brainstem pathways.

The main goal of this study is to determine if the efficacy of CPM can be manipulated by pharmacologically enhancing and reducing autonomic responses during CPT. We hypothesized that enhancing sympathetic nervous activity with a sympathomimetic drug (ephedrine, *α,* and *β*-adrenergic agonist) during the CPT would increase CPM effectiveness in healthy individuals. Conversely, we hypothesized that inhibiting sympathetic activity during CPT with a *β*-blocker (esmolol, selective *β*1-blocker) would decrease CPM efficiency. The objective of this study was to describe the effects of pharmacologic enhancements (sympathomimetic) and reductions (sympathetic antagonist) of autonomic nervous system activity on the CPM in healthy participants.

## 2. Methods

### 2.1. Study Participants

A convenience sample of 30 healthy volunteers participated in the study. Sample size was calculated using previous data obtained in our lab with an anticipated effect size (Cohen's *d*) estimated at 0.66, a desired statistical power level fixed at 80% and a probability level of 0.05 [25]. All 30 participants were healthy adults (men and women, aged between 18 and 45 years) and were recruited in the city of Sherbrooke using a snowball sampling method. Exclusion criteria were cardiovascular disease (coronary artery disease, heart failure, arrhythmia, hypertension, etc.), asthma, any pain condition (acute or chronic), endocrine conditions (hyperthyroidism, diabetes, etc.), neurologic or psychiatric pathology, and usage of any medication (or illicit drug) considerably affecting the autonomic nervous system (*β*-blockers, decongestants, etc.). Pregnant or breastfeeding women were also excluded. Patients were instructed to refrain from using short-term analgesics (acetaminophen, ibuprofen, etc., for acute pain, headache, and fever) at least 24 hours prior to testing and to abstain from using caffeine or nicotine 4 hours before experimental sessions. All participants signed a written consent form, and all the procedures were approved by the Human Ethics Committee of the Centre Hospitalier Universitaire de Sherbrooke (CHUS; project # 10-152).

Of the thirty individuals included in this study, 26 participants completed the entire experimental procedure. One individual was removed from all the analyses due to technical problems related to the recording of the data. One male participant presenting sinus bradycardia at rest (≤50–55 beats per minute) was ineligible for the esmolol session (contraindicated for *β*-blockers usage) and excluded from the analyses. All other experimental sessions of this individual were included in the final analyses. Also, two other participants (one male and one female) were not included in the main CPM analyses because of the absence of baseline CPM values.

### 2.2. Experimental Procedures

The protocol included four sessions (performed on four different days) consisting of experimental pain tests used to evaluate the effectiveness of CPM. The first experimental session was a baseline evaluation (no medication or placebo) of CPM efficacy (difference in pain intensity during the heat-test stimuli performed before and after the CS). During the three following sessions, an anesthesiologist administered a 10 ml syringe including (1) esmolol (0.5 mg/kg), (2) ephedrine (15 mg), or (3) placebo (5 ml lactate ringer) in a 0.9% NaCl solution. These three visits were performed in a randomized order (randomization sequence was determined with a computerized generated randomization table). Medication and placebo were prepared by the hospital pharmacy of the clinical research center of the CHUS. Medications were given by the same anesthesiologist for all participants using an intravenous line (22-gage needle) inserted in the left antecubital vein near the elbow on the right arm 2 minutes before the CPT. Participants and the research associates performing the experimental pain tests were blinded to drug administration. However, the anesthesiologist was not blinded for safety reasons (to optimize emergency procedures in case an adverse event occurred).

Participants completed questionnaires evaluating physiological and psychological characteristics during the first experimental visit. During all experimental sessions, participants were seated in a quiet room. The participants were familiarized with the experimental heat pain test delivered with a 3 cm^2^ thermode (TSA II, Medoc Advanced Medical Systems, Ramat Yishai, Israel) applied on the volar side of the left forearm. Heat pain threshold and heat pain tolerance were first evaluated. Then, the heat-test stimuli were performed before and immediately after the CPT (CS) to evaluate CPM effectiveness at each visit.

### 2.3. Questionnaires

At the first experimental session, the French versions of the questionnaires were completed by all participants. The State and Trait Anxiety Inventory (STAI) was used to assess state and trait of anxiety [26, 27], the Beck Depression Inventory (BDI) was used to evaluate depressive mood [28, 29], and the Pain Catastrophizing Scale (PCS) was used to assess pain catastrophizing thinking [30, 31]. State anxiety was also assessed at the beginning of all experimental sessions.

### 2.4. Pain Measurements

Pain intensity during the experimental heat pain test stimulation was continuously evaluated with a computerized visual analog scale (CoVAS) ranging from 0 (no pain) to 100 (most intense pain tolerable). During the CPT, pain intensity was verbally measured with a numeric rating scale (0: no pain to 100: most intense pain tolerable).

### 2.5. Heat Pain Threshold and Heat Pain Tolerance

A thermode was initially set at 32°C and increased at a rate of 0.3°C/sec. Participants were instructed to use the CoVAS to continuously evaluate their pain perception. More specifically, they were asked to report when the heat sensation changed from a hot sensation to a mild pain sensation (1/100 on the CoVAS; heat pain threshold) and when the pain intensity became unbearable (100/100; heat pain tolerance). This procedure was performed twice for each participant (or until the values were constant ≤0.5°C). The means of these 2 procedures were calculated to determine the participants' heat pain threshold and heat pain tolerance. The thermode was placed on different parts of the forearm for every test to avoid skin hyperalgesia. The thermode temperature causing moderate pain during this procedure was used to perform the subsequent heat-test stimuli.

### 2.6. Heat-Test Stimulus

During the heat-test stimulus, the thermode was applied to the left forearm (volar part) for 120 sec. Participants continuously rated their pain intensity on the CoVAS throughout the entire heat-test stimulus (scores were generated on the computer every 0.05 sec). The temperature used for the heat-test stimulus was individually adjusted to cause moderate pain (approximately 50/100 pain intensity on the CoVAS, evaluated during the heat pain threshold and tolerance procedures). Participants were instructed that the temperature of the thermode could change (increase, decrease, or remain stable) during the heat-test stimulus to reduce expectations (to avoid bias in participants' responses). In fact, after a rise from 32°C (at a rate of 0.3°C/sec), the temperature of the thermode remained stable (pain 50, individually adjusted thermode temperature) during the entire 120 sec [32–35]. The heat-test stimulus was performed before and after the CPT (CS) using the same individually determined thermode temperature. Pain intensity during the heat-test stimulus is represented by the mean of all scores registered by the CoVAS during the 120 sec of stimulation.

### 2.7. Cold Pressor Test (CPT)

The CS used to trigger CPM was the cold pressor test (CPT). During CPT, participants had to immerse their right arm (up to the elbow) in cold circulating water (10°C) for 120 sec. Pain intensity induced by the CPT was evaluated at 30 sec intervals with a verbal numeric rating scale. The CPT started 2 minutes after drug/placebo administration.

### 2.8. Cardiovascular Activity

Arterial blood pressure was continuously measured with a Nexfin HD monitor (BMEYE, Amsterdam, Netherlands) applied to a finger of the left hand (index or middle finger). Nexfin is a noninvasive device that was validated compared to conventional measurement of blood pressure. [36] Electrocardiographic (ECG) activity was recorded using a 3-electrode system (AD instruments, Colorado Springs, CO, USA) to continuously evaluate the heart rate. Cardiac activity monitoring allowed the anesthesiologist to rule out bradycardia (or tachyarrhythmia) at the beginning of the experimentation. Cardiovascular activity (mean heart rate and blood pressure) was evaluated at baseline (2 min) and during the CPT (2 min).

### 2.9. Statistical Analysis

All statistical analyses were conducted using SPSS version 28 (IBM Corp, Armonk, NY, USA) and SAS version 9.4 (SAS Institute Inc., Cary, NC, USA). The recommendations for analyzing CPM were followed [7]. CPM scores were obtained by calculating the percentage of the difference between the heat-test stimulus pain score (i.e., mean pain intensity obtained during the two-min thermode test) before and after the conditioning stimulus (CPT). CPM was considered effective when heat-test stimulus pain intensity was experienced as less painful after the CPT compared to before (i.e., % change of mean pain intensity from before to after CPT <0).

A comparison of physiological and psychological characteristics at baseline was made between men and women using Student's *T*-test. Pain intensity ratings before and during CPT, heart rate, and mean arterial blood pressure were compared using mixed models including conditions (baseline, esmolol, ephedrine, and placebo), gender, and the interaction between those two variables (it was removed if not significant) with a random intercept by participants. Gender differences were evaluated using contrasts in the same model. Multiple comparisons for conditions were adjusted using the Tukey–Kramer method. CPM differences from baseline (delta) were compared using a mixed model with gender, postconditions (esmolol, ephedrine, and placebo), and the interaction between those two variables, in addition to the baseline CPM value with a random intercept by participants. Gender and condition differences were evaluated using contrasts in the same model. Mixed models were preferred to ANOVA or ANCOVA because of the presence of a small number of missing values. Data are expressed as a mean and standard error of the mean (SEM). Statistical significance was set at *p* < 0.05 (two-tailed).

## 3. Results

### 3.1. Characteristics of the Participants

Physiological (age, height, weight) and psychological (trait anxiety, depressive symptoms, and pain catastrophizing) characteristics of the participants are presented in Table 1. Student's *T*-tests for independent samples revealed that in our sample, men were slightly older, taller, and heavier than women (all *t* values >2.33, all *p*-values <0.05). Trait anxiety was slightly higher in women than in men (*t* value = −2.07, *p*=0.024).

### 3.2. Pain Intensity Ratings before CPT

Heat pain threshold, heat pain tolerance, pain-50 evaluations, and pain intensity during the heat-test stimuli with the thermode before the CPT for men and women in the different conditions are shown in Table 2. We did not observe any significant difference across sex or conditions for any of our pain intensity ratings (heat pain threshold/tolerance or pain-50 evaluations; all type III *F* values <3.89, all *p* values ≥0.05). Importantly, pain intensity during the heat-test stimulus performed before the CPT was comparable for different genders and conditions (type III *F* gender = 0.02, *p* gender = 0.88, type III *F* condition = 0.21, *p* condition = 0.89, type III *F* interaction = 0.91, and *p* interaction = 0.44).

### 3.3. Pain Intensity Ratings during CPT

**Figure 1:**
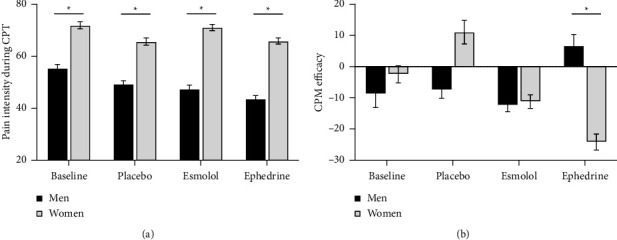
Scores for men and women for the 4 different conditions (baseline, placebo, esmolol, and ephedrine). (a) Mean pain intensity ratings during the cold pressor test (CPT). (b) Mean conditioned pain modulation (CPM) effectiveness. ^*∗*^*p* < 0.05 for the difference between men and women calculated using contrast in the overall mixed model that includes the interaction between condition and sex.

The mean pain intensity scores during the CPT for the 4 different conditions in men and women are presented in Figure 1(a). Pain intensity was lower in men than in women for all conditions (all type III *F* values ≥5.46, all *p* values <0.05). Compared to baseline, pain intensity was lower during the placebo and ephedrine conditions (*t* value placebo = 3.30, *p*_adj_ placebo = 0.008, *t* value ephedrine = 4.76 and *p*_adj_ ephedrine <0.0001) with no sex × condition interaction (type III *F* value = 1.76, *p*=0.16).

### 3.4. Conditioned Pain Modulation (CPM)

The effectiveness of CPM for the 4 different conditions in men and women is presented in Figure 1(b). The effectiveness of CPM was comparable in different conditions (type III *F* = 1.34, *p*=0.3) and gender (type III *F* = 0.51, *p*=0.5). However, a statistically significant sex × condition interaction was found (type III *F* = 3.58, *p*=0.036). CPM effectiveness change from baseline was higher in women than in men during the ephedrine condition (*t* = −2.42, *p*=0.019), and CPM effectiveness change from baseline was higher during the ephedrine condition than placebo but only in women (*t* = −2.60, *p*=0.012).

### 3.5. Cardiac Activity

The mean heart rate at rest, during CPT, and delta (CPT-rest) heart rate values for the different conditions in men and women are presented in Table 3. The sex × condition interactions were not significant (rest, during CPT, or delta scores; all type III *F* < 2.57, all *p* values >0.05). However, we observed significant condition differences and sex effects. There were significant gender differences for HR before CPT, HR during CPT, and HR delta (type III *F* before = 5.62, *p* before = 0.025, type III *F* CPT = 13.85, *p* CPT = 0.0009, type III *F* delta = 16.16, *p* delta = 0.0004). There was a significant condition difference for HR during CPT (all pairs were significant except the baseline-placebo pair, *t* baseline-ephedrine = 3.37, *p* baseline-ephedrine = 0.001, *t* baseline-esmolol = −2.16, *p* baseline-esmolol = 0.03, *t* placebo-ephedrine = 3.44, *p* placebo-ephedrine = 0.0009, t placebo-esmolol = −2.02, *p* placebo-esmolol = 0.046, *t* ephedrine-esmolol = −5.49, *p* ephedrine-esmolol <0.0001) and the HR delta (all pairs were significant except the baseline-placebo pair, *t* baseline-ephedrine = 2.94, *p* baseline-ephedrine = 0.004, *t* baseline-esmolol = −3.43, *p* baseline-esmolol = 0.001, *t* placebo-ephedrine = 3.76, *p* placebo-ephedrine = 0.0003, *t* placebo-esmolol = −2.56, *p* placebo-esmolol = 0.01, *t* ephedrine-esmolol = −6.34, *p* ephedrine-esmolol <0.0001).

### 3.6. Blood Pressure

The mean arterial blood pressure (MAP) at rest, during CPT, and blood pressure variations (delta: CPT-rest) are presented in Table 4 for the different conditions in men and women. The sex × condition interactions were not significant (rest, during CPT or delta scores; all type III *F* < 0.59, all *p*-values *>*0.05). However, we observed significant condition differences and sex effects. There were significant gender and condition differences for MAP at rest (type III *F* = 11.07, *p* gender = 0.003, baseline was significantly different from ephedrine and esmolol with *t* = −3.24 and *p*=0.0018 and *t* = −2.89 and *p*=0.005, respectively). There was also a significant condition difference for the MAP delta (ephedrine was significantly different from all 3 conditions with *t* baseline = 2.79, *p* baseline = 0.007, *t* placebo = 3.78, *p* placebo = 0.0003, *t* esmolol = 2.44 and *p* esmolol = 0.017).

## 4. Discussion

The objective of this randomized, double-blind, placebo-controlled investigation was to determine the effects of pharmacological drugs affecting cardiovascular activity on the effectiveness of endogenous pain inhibition (CPM) in healthy individuals. Our main finding was that ephedrine (*α* and *β*-adrenergic agonist) importantly increased the effectiveness of CPM in women, whereas this effect was inhibited in men. Dayan and colleagues performed an interesting study comparing the effectiveness of CPM in healthy participants after administration of phenylephrine (*α*-adrenergic agonist), clonidine (selective *α*_2_ agonists), yohimbine (*α*_2_ agonists, also interacting with adrenergic, serotonergic, and dopaminergic receptors), and saline. The authors reported that autonomic modulation with these drugs did not have any significant effect on CPM effectiveness [37]. However, sex-dependent effects of these drugs on CPM were not investigated in this study. Importantly, *α*_2_ agonists, such as clonidine, inhibit sympathetic outflow (autoreceptor) and have been shown to have potential analgesic effects [38, 39]. However, the analgesic effect of *α*_2_ agonists (clonidine and dexmedetomidine) had variable effects on conditioned pain modulation effectiveness in healthy individuals [40, 41]. Drugs acting on *α* and *β*-adrenergic receptors could also have different effects on CPM effectiveness by activating/inhibiting various brainstem regions (periaqueductal gray, nucleus raphe magnus, etc.). For instance, the injection of phenylephrine close to the nucleus raphe magnus significantly blocked descending endogenous pain inhibition in male rats [42].

Antagonizing *β*-receptors have been shown to have an analgesic effect in women suffering from fibromyalgia and temporomandibular disorder [43, 44]. However, our results do not suggest *β*-blockers (esmolol) have significant effects on pain intensity (during the CPT) or CPM effectiveness in healthy men or women. Reducing *β*-receptors activation could have beneficial effects in women suffering from chronic pain, but these pain inhibitory effects do not seem to depend on endogenous pain modulation mechanisms such as CPM. The analgesic effects of *β*-blockers reported in chronic pain patients could be attributable to other (peripheral, spinal, or supraspinal) mechanisms and should be investigated in future studies.

Several investigators observed that healthy women have less effective CPM than men [9, 16–19]. Contradictory results of comparative effectiveness of endogenous pain inhibition being equivalent in men and women have also been reported [21, 22]. In the present study, CPM efficacy was comparable between healthy men and women for all conditions (baseline, placebo, and esmolol), except in the ephedrine condition. The presence of sex differences in CPM effectiveness remains a controverted matter. Sex differences in CPM effectiveness could be attributable to sex hormones (estrogen and progesterone) [20, 45]. Various different biopsychosocial factors, such as autonomic activity, could explain sex differences in endogenous pain modulatory mechanisms such as CPM [19, 22, 46].

Greater increases in blood pressure during the CS have been shown to be associated with more effective CPM in healthy individuals [25]. Moreover, women suffering from fibromyalgia have less efficient CPM and show lower blood pressure increases during CS than healthy participants [11]. Higher cardiac parasympathetic activity (root mean square of successive differences RMSSD) has been correlated to CPM effectiveness in men but not in women [19]. Autonomic activity seems to be linked with activation of CPM, although this relationship could be influenced by sex. Interestingly, the gender-specific increased CPM efficacy by the sympathomimetic ephedrine was not related to autonomic activity. The ephedrine effect on CPM may then be related to other central nervous effects such as action on noradrenergic receptors that could act directly on CPM [47].

Psychological factors such as anxiety [48] or catastrophizing [49] have been demonstrated to change brain-related activity for pain perception and endogenous pain modulation. When we adjusted our mixed model analysis for baseline anxiety and catastrophizing, we found no significant effects of these variables. It would be of interest to test potential interactions of these variables in a larger group to have more statistical power.

Improving our understanding of the interaction between autonomic activity, gender, and CPM could lead to improved pain management strategies. Specific therapeutic interventions influencing sympathetic (or parasympathetic) activity could be developed to increase CPM efficiency. More specifically, our results suggest that sympathomimetics, such as phenylephrine, could boost CPM effectiveness in women but not in men. These treatments may be used to relieve chronic pain patients showing weakened endogenous pain inhibition and to optimize the efficacy of some analgesic medications, such as duloxetine [7].

### 4.1. Limitations

This study was realized with healthy individuals, but results may be different in patients suffering from chronic pain. The administration of medication (ephedrine, esmolol, or placebo) was performed at the beginning of the experimental sessions, given the relatively short half-life of these drugs, most of the pharmacologic effects had worn off after the CPT. Esmolol administration was weight-adjusted, while identical doses of ephedrine were given to all participants. Given the fact that women had significantly lower body weight than men, lower ephedrine doses administered (per kg) to men could have influenced our results (considering the relatively short half-life of ephedrine).

For security reasons, the established dose of esmolol was set at 0.5 mg/kg. This dose could have been too low to cause an effect on CPM effectiveness as suggested by the weak cardiovascular responses observed in this study.

Even if the study had a good double-blind method, participants could have felt a change in their heart rate with esmolol and ephedrine. Even if we got no information from the subject suggesting this effect, we did not systematically verify the efficacy of the participant's blinding.

## 5. Conclusions

In summary, the main outcome emerging from this randomized, double-blind, placebo-controlled investigation is that the magnitude of endogenous pain inhibition is affected by *α* and *β*-adrenergic agonists, such as ephedrine. The effectiveness of CPM is greater in healthy women than in men after administration of ephedrine. Our results suggest that sympathomimetics, such as ephedrine, could improve CPM effectiveness in women, whereas this effect seems to be blocked in men.

## Figures and Tables

**Table 1:** Physiological and psychological characteristics of the participants.

Variables	Men	Women	*t* values from Student's *T*-test	*p* value from Student's *T*-test
*N*	14	15		
Age (years)	30.5 ± 1.3	26.5 ± 1.1	2.337	0.014
Height (cm)	181.0 ± 1.7	164.1 ± 1.6	7.258	<0.001
Weight (kg)	83.0 ± 2.7	65.9 ± 3.2	4.005	<0.001
Trait anxiety (STAI) (range 0–80)	6.6 ± 1.4	11.1 ± 1.7	−2.070	0.024
Beck depression inventory (range 0–63)	1.1 ± 0.4	2.8 ± 1.1	−1.420	0.083
Pain catastrophizing scale (range 0–52)	5.4 ± 1.2	8.8 ± 1.7	−1.652	0.055

Data are presented in mean ± SEM. SEM, standard error of the mean; STAI, state and trait anxiety inventory.

**Table 2:** Mean pain evaluations for men and women before CPT.

		Baseline	Placebo	Esmolol	Ephedrine
Men (*n* = 14)	Pain threshold (°C ± SEM)	43.2 ± 1.0	42.4 ± 0.8	42.1 ± 0.8	42.4 ± 0.9
	Pain tolerance (°C ± SEM)	49.7 ± 0.3	49.5 ± 0.3	49.7 ± 0.3	49.4 ± 0.3
	Pain-50 (°C ± SEM)	47.2 ± 0.3	46.9 ± 0.4	47.2 ± 0.3	47.0 ± 0.3
	Pain intensity during the heat-test before CPT (0–100)	52.4 ± 6.1	47.5 ± 3.4	50.1 ± 3.9	45.6 ± 3.5

Women (*n* = 15)	Pain threshold (°C ± SEM)	43.5 ± 0.5	43.6 ± 0.6	43.2 ± 0.7	43.6 ± 0.6
	Pain tolerance (°C ± SEM)	48.5 ± 0.4	48.8 ± 0.3	48.6 ± 0.5	48.7 ± 0.4
	Pain-50 (°C ± SEM)	46.4 ± 0.4	46.7 ± 0.3	46.6 ± 0.3	46.6 ± 0.4
	Pain intensity during the heat-test before CPT (0–100)	48.3 ± 5.8	46.5 ± 5.3	45.2 ± 4.4	51.9 ± 6.1

SEM, standard error of the mean; CPT, cold pressor test.

**Table 3:** Mean heart rate for the different conditions in men and women.

		Baseline (BPM ± SEM)	Placebo (BPM ± SEM)	Esmolol (BPM ± SEM)	Ephedrine (BPM ± SEM)
Men (*n* = 14)	Rest	68.5 ± 2.5	65.7 ± 2.1	71.0 ± 1.8	65.9 ± 2.1
	CPT	71.1 ± 1.8	68.5 ± 2.1	68.3 ± 1.3	72.5 ± 2.1
	Delta	2.6 ± 1.0	2.8 ± 1.1	−2.7 ± 1.3	6.6 ± 1.2

Women (*n* = 15)	Rest	73.4 ± 3.3	77.9 ± 4.1	74.9 ± 3.1	77.8 ± 3.5
	CPT	81.0 ± 3.1	83.9 ± 3.1	78.3 ± 3.4	89.4 ± 3.9
	Delta	7.6 ± 1.7	5.1 ± 1.7	3.4 ± 1.3	11.6 ± 1.9

SEM, standard error of the mean; BPM, beats per minute; CPT, cold pressor test; Delta: CPT-rest.

**Table 4:** Mean arterial blood pressure for men and women.

		Baseline (mmHg ± SEM)	Placebo (mmHg ± SEM)	Esmolol (mmHg ± SEM)	Ephedrine (mmHg ± SEM)
Men (*n* = 14)	Rest	106.5 ± 3.1	100.1 ± 2.5	97.7 ± 3.2	97.2 ± 2.2
	CPT	124.6 ± 3.9	115.2 ± 3.3	117.5 ± 5.0	121.2 ± 3.1
	Delta	18.1 ± 2.4	15.0 ± 2.7	19.8 ± 4.4	23.9 ± 2.2

Women (*n* = 15)	Rest	95.3 ± 2.3	94.9 ± 2.2	90.7 ± 2.5	89.4 ± 2.9
	CPT	113.8 ± 3.5	111.4 ± 4.1	109.1 ± 3.1	114.5 ± 4.2
	Delta	18.5 ± 3.2	16.0 ± 2.8	18.4 ± 2.3	25.3 ± 2.7

SEM, standard error of the mean; CPT, cold pressor test; Delta: CPT-rest.

## Data Availability

The data that support the findings of this study are available upon request from the corresponding author.
